# Differential regulation of MMP activity by TGFβ1 in fast- and slow- twitch muscle repair: insights from EDL and soleus muscle-derived myoblasts

**DOI:** 10.3389/fcell.2025.1592512

**Published:** 2025-06-04

**Authors:** Paulina Kasprzycka, Maria Anna Ciemerych, Malgorzata Zimowska

**Affiliations:** Department of Cytology, Faculty of Biology, University of Warsaw, Warsaw, Poland

**Keywords:** TGFβ1, matrix metalloproteinases, MMP-2 and MMP-9, skeletal muscle, satellite cells

## Abstract

**Introduction:**

Skeletal muscles are characterized by a significant ability to regenerate in response to injury. However, muscle repair is often inefficient and hindered by the development of fibrosis. The course of muscle repair is related to the type of skeletal muscle, i.e., fast- versus slow-twitch, and is controlled by various factors. Among them are TGFβ1 and two MMPs, i.e., MMP-2 and MMP-9 gelatinases that play a key role in the remodeling of the extracellular matrix (ECM). Although the role of TGFβ1 in the regulation of ECM protein synthesis is well established, its involvement in the regulation of enzymes, such as MMPs, is still not well understood. In this study, we investigated the relationship between TGFβ1 and MMP-9/MMP-2 in *in vitro* differentiating myoblasts isolated from rat slow-twitch Soleus or fast-twitch Extensor Digitorum Longus (EDL) muscles. We hypothesized that differences in the regulation of MMPs contribute to the varying repair efficiencies between muscle types.

**Methods:**

Using siRNA to silence TβR1 expression, suramin as a competitive inhibitor of the TβR1 receptor, and inhibitors of both the canonical and non-canonical TGFβ signaling pathways, we characterized the role of TGFβ1 in regulating MMP-9 and MMP-2 during differentiation of myoblasts derived from slow-twitch Soleus and fast-twitch EDL muscles *in vitro*.

**Results and discussion:**

Our results demonstrated that blocking TGFβ1 signaling pathway significantly improved regeneration in slow-twitch Soleus muscle, altered the activity of MMP-9 and MMP-2 in *in vitro* differentiating myoblasts, and Soleus and EDL-derived myoblasts differ in their response to inhibition of TGFβ-dependent signaling pathways.

## Introduction

Skeletal muscles are characterized by an ability to regenerate in response to injury or disease (Charge and Rudnicki, 2004; Laumonier and Menetrey, 2016). Upon muscle injury, satellite cells are activated, forming a population of myoblasts that proliferate, fuse and form multinucleated myotubes and myofibers, replacing the destroyed ones. Myoblast differentiation remains under the control of transcription factors that belong to the MRF (Myogenic Regulatory Factors) family, that is, MyoD, Myf5, myogenin, and MRF4. This process is also strongly associated with the reconstruction of extracellular matrix (ECM) (Motohashi and Asakura, 2014; Costamagna et al., 2015; Dumont et al., 2015). Among the enzymes involved in ECM restoration are matrix metalloproteinases (MMPs), a family of multidomain zinc and calcium-dependent endopeptidases that can selectively hydrolyze such ECM components as collagen, gelatin, elastin, proteoglycan core proteins, and fibronectin. MMPs not only degrade ECM proteins, creating space for cells to migrate, but also impact cell proliferation, death, and differentiation (Chen and Li, 2009; Apte and Parks, 2015; Singh et al., 2015). Their action is regulated at different levels, including post-transcriptional, i.e., activation of latent proenzyme forms or interactions with endogenous inhibitors (Piperi and Papavassiliou, 2012; Gaffney et al., 2015). The major MMP inhibitors are TIMPs (MMP tissue inhibitors), whose family is composed of four members: TIMP1, TIMP1,-2,-3, and -4. MMP activity may also be regulated by nonspecific inhibitors, e.g., α2-macroglobulin, thrombospondin-1, thrombospondin-2 or RECK (reversion-inducing cysteine-rich protein with Kazal motifs) (Alameddine, 2012; Arpino et al., 2015; Gaffney et al., 2015).

In skeletal muscle, two MMPs, that is, MMP-2 and MMP-9, play a key role in the function of the ECM (Chen and Li, 2009; Alameddine, 2012). Under physiological conditions, their activity is low but increases during muscle repair or remodeling that accompanies injury or the development of disease (Kherif et al., 1999; Zimowska et al., 2008). In injured muscle, during the myolysis phase, MMP-9 activity predominates, as its main function is to degrade ECM proteins and facilitate myoblast proliferation and migration. MMP-2 activity becomes dominant in the reconstruction phase and promotes the adjustment of the cellular environment to facilitate the development of new muscle fibers (Chen and Li, 2009; Apte and Parks, 2015).

Our previous study showed that the expression and activity of MMP-9 and MMP-2 depend on the type of skeletal muscle (Zimowska et al., 2008). Fast- twitch muscles, such as Extensor Digitorum Longus (EDL), contain 95% of fast fibers, while slow-twitch muscles, such as Soleus, contain 80–100% of slow fibers (Schiaffino and Reggiani, 2011; Schiaffino, 2018). After injury, the EDL muscle regenerates properly, while in Soleus damaged muscle fibers are replaced by connective tissue. In the regenerating EDL muscles, the MMP-9 level decreases during myolysis, while MMP-2 activity increases during the reconstruction phase. In the slow-twitch Soleus muscle, high MMP-9 activity accompanies both myolysis and reconstruction phases (Zimowska et al., 2008). Importantly, inhibition of MMP-9 activity results in a significant improvement of Soleus regeneration, i.e., restriction of ECM protein deposition and fibrosis formation (Zimowska et al., 2012). Thus, understanding the mechanisms of MMP control is a prerequisite for explaining the differences between fast- and slow-twitch skeletal muscle repair.

Skeletal muscle regeneration is controlled by various growth factors or cytokines. Many lines of evidence document that TGFβ (transforming growth factor beta) family members can play a key role in this process. They are known as stimulators and modulators of the synthesis of ECM components, ECM-degrading enzymes, and their inhibitors (Shi and Massague, 2003; Kubiczkova et al., 2012; Weiss and Attisano, 2013). In skeletal muscles, one of the TGFβ family members, e.g., TGFβ1, plays a key role in controlling tissue remodeling accompanying repair (MacDonald and Cohn, 2012; Mendias et al., 2012; Sartori et al., 2014). It also negatively regulates myoblast proliferation and differentiation (Massague et al., 1986; Hathaway et al., 1991; Li et al., 2004; Carlson et al., 2009; Cohen et al., 2015). TGFβ1 signaling is primarily mediated through two distinct pathways: the canonical (Smad-dependent) and non-canonical (Smad-independent) pathways. In the canonical pathway, TGFβ1 binds to its type II receptor (TβR2), which recruits and phosphorylates the type I receptor (TβR1). Such activation leads to the phosphorylation of receptor-regulated Smad proteins (R-Smads: Smad2 and Smad3). Phosphorylated R-Smads then form a complex with the common mediator Smad (Co-Smad, Smad4), which translocate to the nucleus to regulate the transcription of target genes. The non-canonical pathways include multiple signaling cascades activated by TGFβ1 independently of Smad proteins: MAPK pathways (ERK, JNK, p38), PI3K/AKT pathway, Rho-Like GTPases or NF-κB pathway (Weiss and Attisano, 2013).

Our previous study demonstrated that the levels of TGFβ1 and TβR1 (TGFβ receptor type 1) changed during the *in vitro* differentiation of myoblasts isolated from the Soleus and EDL muscles, as well as during the regeneration of these muscles. Furthermore, inhibition of TGFβ1 or TβR1 function resulted in a significant improvement of Soleus muscle regeneration (Zimowska et al., 2009) which could be connected to the role of TGF1 as a factor modifying the synthesis and proteolysis of ECM components. Such an impact on the MMP could be mediated by regulation of their expression and/or activity. Thus, it is possible that the differences between fast- and slow-twitch muscle regeneration and differentiation of myoblasts isolated from these muscles are caused by different ‘relationships’ between TGFβ1 and MMPs.

The purpose of the current study was to analyze the interplay between TGFβ1 and MMP-2, as well as MMP-9. We hypothesized that differences in fast- and slow-twitch muscle repair may result from variations in the regulation of MMP activity. To test this, we inhibited the TGFβ1 signaling pathway in *in vitro* differentiating myoblasts isolated from rat slow-twitch Soleus and fast-twitch EDL muscles. Using siRNA to silence TβR1 expression, suramin as a competitive inhibitor of the TβR1 receptor, and inhibitors of both the canonical and noncanonical TGFβ1 signaling pathways, we characterized the role of TGFβ1 in the regulation of MMP-9 and MMP-2 during differentiation of myoblasts derived from slow-twitch Soleus and fast-twitch EDL muscles *in vitro*.

## Materials and methods

### Induction of muscle regeneration

Soleus and EDL muscle regeneration was induced in 3-month-old male Wistar rats following established protocols (Lagord et al., 1998). Rats were anesthetized with an intraperitoneal injection of ketamine (60 mg/kg) and xylazine (6 mg/kg). The target muscle was exposed, the tendons preserved, and the motor nerve severed at the muscle surface. The muscle was then crushed in its entirety using a Pean hemostatic forceps and repositioned. Following skin closure, the animals were returned to their cages with *ad libitum* access to food and water. This standardized procedure consistently induced extensive muscle fiber damage, facilitating subsequent biochemical analyses. To investigate the role of TGFβ1, antibodies against TGFβ-receptor I (Santa Cruz) were injected (10 μg per muscle in 50 μL) immediately after crush into the designated muscle. Muscles injected with NaCl were used as a control. On different days after injury (days 1, 3, 7, and 14), the animals were sacrificed using CO_2_, and the regenerating muscles were removed. Each experimental group included three rats, and the entire experiment was replicated three times.

### Isolation and culture of satellite cell-derived myoblasts

Satellite cells were isolated from intact Soleus and EDL muscles of 3-month-old Wistar male rats, as previously described (Zimowska et al., 2005). Satellite cells were isolated by digestion with 0.15% pronase (Sigma) for 1.5 h in Ham F12 medium buffered with 10 mM HEPES containing 10% fetal calf serum (FCS). Next, the digested tissue was filtered and then centrifuged three times for 20 min at 20,000 rpm. 30,000 cells/cm^2^ were seeded in 35–mm–diameter dishes (Becton, BD Bio Sciences) coated with 3% gelatin (Sigma) and continuously cultured in Dulbecco’s modified Eagle’s medium containing 10% fetal bovine serum and 10% horse serum (Gibco, Invitrogen Ltd.) in 5% CO_2_ at 37°C.

### siRNA treatment

Soleus or EDL derived myoblasts were either transfected with 8 nM Stealth siRNA Negative Control siRNA (Thermo Fisher Scientific) or 8 nM Stealth siRNA predesigned siRNA (Thermo Fisher Scientific) complementary to TβR1. The sequences of the TβR1 siRNAs were: 5′-GGACCAUUGUGCUACAAGAtt-3′ (sense) and 5′-UCUUGUAGCACAAUGGUCCtt-3′ (antisense). Untreated myoblasts or myoblasts transfected with 8 nM Stealth siRNA Negative Control siRNA served as a control. When the cells reached 50%–70% confluency (day 5 of culture - proliferation stage), they were transfected with the appropriate siRNA using the Lipofectamine 2000 transfection reagent (Thermo Fisher Scientific), according to the manufacturer’s instructions. The efficiency of transfection and silencing of TβR1 mRNA expression was analyzed by qPCR at 48 and 72 h after treatment, corresponding to days 7 and 8 of culture. Compared to the control, expression was reduced by 69% and 42% in EDL-derived myoblasts and 82% and 46% in Soleus-derived myoblasts 48 and 72 h after transfection, respectively. Additionally, we confirmed the absence of TβR1 protein in EDL-derived myoblasts 72 h after transfection and in Soleus-derived myoblasts 48 h after transfection with siRNA complementary to TβRI (Western blot analysis).

### Inhibitors treatment

At 5 days of *in vitro* culture, myoblasts were treated with suramin (50 µg/mL) (competitive inhibitor of the TβR1 receptor), 0.5 nM SIS3 (Smad3 phosphorylation inhibitor), 0.5 nM halofuginone (Smad7 activator, Smad3 phosphorylation inhibitor) (Sigma), 5 μM U0126, 5 μM PD98059 (MEK1 inhibitor), and 1 μM SB202190 (p38 MAP kinase inhibitor) (Abcam). Cells were analyzed 48 or 72 h after treatment, corresponding to days 7 and 8 of culture. The control myoblasts were cultured under standard conditions.

### Index of fusion

At 48 or 72 h after treatment (corresponding to days 7 and 8 of culture), control and experimental myoblasts were stained with May–Grünwald–Giemsa stain (Merck) for myotube classification and fusion index determination. The fusion index was calculated as the percentage of nuclei within myotubes relative to the total number of nuclei in the field of view. At least ten representative microscopic fields were analyzed per culture. Each experiment was repeated three times.

### qPCR

RNA was isolated from control and treated Soleus or EDL-derived myoblasts. RNA isolation was performed using the MirVana PARIS Isolation Kit (Thermo Fisher Scientific) and then treated with TURBO DNase (Thermo Fisher Scientific). Reverse transcription was performed using 0.5 μg total RNA and the RevertAid First Strand cDNA Synthesis Kit (Thermo Fisher Scientific), according to the manufacturer’s instructions. qPCR was performed using the following specific TaqMan® probes: Rn00579162_m1 (MMP-9), Rn01538170_m1 (MMP-2), Rn00562811_m1 (TβR1), and Rn01775763_g1 (GAPDH), using the TaqMan Gene Expression Master Mix (Thermo Fisher Scientific) and the Light Cycler 96 instrument (Roche). Data were collected and analyzed with Light Cycler 96 SW1.1 software (Roche). Analysis of relative gene expression using quantitative PCR and the 2^-Delta Delta Ct method was performed according to Livak and Schmittgen (Livak and Schmittgen, 2001).

### Immunostaining and *in situ* zymography

The samples were washed in PBS and permeabilized in 0.05% Triton X-100 (Sigma) in PBS, washed in PBS and incubated in 0.25% glycine (Sigma) in PBS, followed by incubation in 3% bovine serum albumin (Sigma) in PBS. The following primary antibodies were used: anti-eMyh (mouse monoclonal Santa Cruz) and anti-laminin (rabbit polyclonal, Sigma), diluted 1:100 in 3% BSA in PBS at 4°C overnight. The samples were then incubated with appropriate secondary antibodies conjugated with Alexa Fluor 488 or 594 (Thermo Fisher Scientific) diluted 1:500 in 1.5% BSA in PBS at room temperature for 2 h. Negative controls using secondary antibodies were performed. Actin filaments were visualized using TRITC-conjugated phalloidin (Sigma). The nuclei were visualized with Draq5 (Biostatus Limited). Gelatinase activity was localized in control or treated regenerated muscles and cultured *in vitro* myoblasts. Detection of enzymatic activity was carried out according to the manufacturer’s instructions (DQ^TM^ gelatin from pig skin, fluorescein conjugate; Molecular Probes). Briefly, samples were incubated with DQ™ gelatin at a final concentration of 2.5 µg/mL diluted in reaction buffer (50 mM Tris-HCl (pH 7.6), 150 mM NaCl, 5 mM CaCl_2_, 0.01% Tween 20) at 37°C for 2 h in the dark. Fluorescence imaging was performed using the LSM 700 confocal microscope (Zeiss) and analyzed with ZEN software (Zeiss). Quantitative analysis of fluorescence intensity was carried out by measuring the mean fluorescence intensity whereas quantitative assessment of fluorophore colocalization was performed using Pearson’s correlation coefficient (PCC), calculated in ZEN software.

### In-gel gelatin zymography

Detection of the enzymatic activity of MMP-2 and MMP-9 was performed by gel gelatin zymography. Control or treated myoblasts were homogenized, mixed with non-reducing sample buffer containing 62.5 mM Tris-HCl, 10% glycerol, 2% SDS, 0.05% bromophenol blue (Sigma) and loaded onto 7.5% SDS-PAGE gels containing 0.1% gelatin (Sigma). After electrophoresis, SDS was removed from the gels by washing them in 2.5% Triton X-100 twice for 20 min. The gels were incubated in buffer containing 50 mM Tris–HCl, 5 mM CaCl2, 200 mM NaCl (Sigma), pH 7.5, and stained with Coomassie blue (Bio-Rad) at room temperature for 48 h. MMP-9 was detected as a band of approximately 98 kDa corresponding to the MMP-9 proenzyme, and the second band was detected at approximately 82 kDa, corresponding to the active form of MMP-9. The MMP-2 proenzyme and activated form were detected as the 68 and 62 kDa bands, respectively.

### Statistical analysis

Statistical analyses were conducted using GraphPad Prism software (version 10.4.0; GraphPad Software, San Diego, CA, United States). The distribution of data was evaluated using the Shapiro–Wilk test to assess normality. For datasets demonstrating a normal distribution (P > 0.05), differences between groups were analyzed using one-way analysis of variance (ANOVA) followed by Holm–Šidák’s *post hoc* multiple comparisons test. For non-normally distributed data (P < 0.05), the nonparametric Kruskal–Wallis test was applied, followed by Dunn’s multiple comparisons test for pairwise group comparisons or the nonparametric Kruskal–Wallis test followed by pairwise comparisons without Dunn’s correction for multiple testing. Statistical significance thresholds and adjusted P value interpretations are provided in the respective figure legends.

## Results

### Impact of TGFβ1 on gelatinases: MMP-9 and MMP-2 activity during muscle regeneration

**FIGURE 1:**
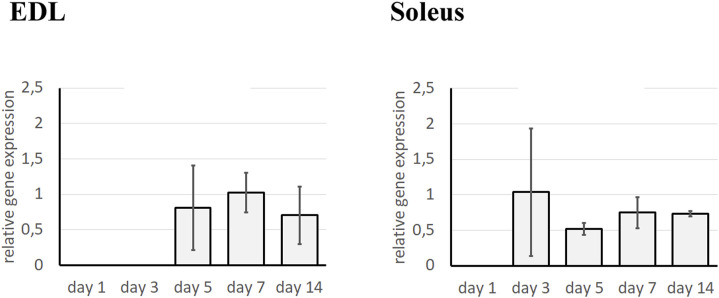
Analysis of TβR1 expression in regenerating Soleus and EDL muscle. Analysis was performed using qRT-PCR at day 1, 3, 5, 7, and 14 after the crush in control (injected with NaCl) muscle. Relative gene expression was calculated relative to the Cq of the reference gene GAPDH. Results are reported as mean ± standard deviation (SD).

Since the enzymes responsible for ECM remodeling are matrix metalloproteinases (MMPs), the effect of the TGFβ1 pathway on the activity of MMP-9 and MMP-2 in fast- and slow-twitch muscles was further investigated. First, we assessed the expression pattern of TβR1 in fast- and slow-twitch muscles (Figure 1). In EDL muscles, no expression of TβR1 was detected up to day 5 of regeneration, reaching a peak on day 7. In contrast, in control regenerating Soleus muscles, TβR1 expression was detected from day 3 of regeneration, remaining at a relatively constant level throughout the analyzed time points. These findings suggest that the dynamics of the TGF-β pathway may differ between fast- and slow-twitch muscles, contributing to their distinct regenerative responses.

**FIGURE 2:**
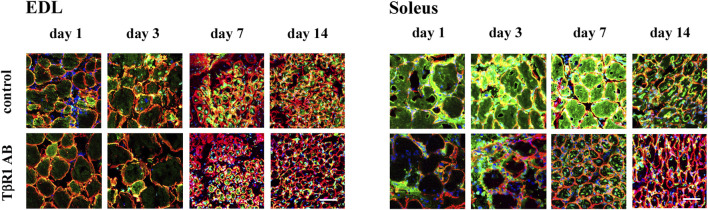
*In situ* zymography of transversal sections of regenerating EDL and Soleus muscles. Gelatinolytic activity was detected at day 1, 3, 7, and 14 after the crush in control (injected with NaCl) or treated with antibody against TGFβ-receptor I (TβR1 AB) muscles. Gelatinolytic activity detected in transversal muscle sections is shown in green, nuclei - blue, laminin - red. Scale bar - 50 μm.

To assess the activity of MMP-9 and MMP-2 gelatinases, *in situ* zymography was employed at day 1, 3, 7, and 14 after injury of Soleus and EDL muscles. This method is commonly used to detect gelatinase activity directly in tissue or cells, without the need for protein extraction; however, it does not differentiate between MMP-9 and MMP-2 activities. The method detects the activity of both gelatinases, as the fluorochrome-conjugated substrate is common to both enzymes. In cross sections of regenerating muscles, active gelatinases were observed within the cytoplasm of damaged fibers, in their surroundings, and within mononuclear cells residing in the injured tissue (Figure 2). On day 1, the signal intensity corresponding to gelatinase activity was lower in the regenerating control EDL muscle compared to the Soleus muscle. Similarly, in the days following muscle injury, the signal observed in EDL muscles remained less intense than that associated with Soleus muscle regeneration. Soleus muscles injected with NaCl (control) exhibited exceptionally high gelatinase activity at the early stage of regeneration (day 1). This was followed by an increase in gelatinase activity on days 3 and 7 after the injury, and a subsequent decrease by day 14 of Soleus muscle repair. Quantitative analysis of gelatinolytic activity showed that treatment of EDL muscles with an anti-TβR1 antibody did not significantly change enzyme activity (Supplementary Figure S1). On the contrary, the Soleus muscles treated with the anti-TβR1 antibody showed a reduced intensity of the gelatinolytic signal compared to the control throughout the repair period. Starting from day 3, the signal in muscles treated with the anti-TβR1 antibody was significantly weaker than in the control group. A similar reduction in signal intensity was observed on day 7. In contrast, by day 14, the signal intensity returned to a level comparable to that of the control (Supplementary Figure S1). Thus, inhibition of the TGFβ1-dependent signaling pathway reduced MMP-9 and MMP-2 activity in regenerating slow-twitch (Soleus) muscles, with a less pronounced effect in fast-twitch (EDL) muscles.

In injured skeletal muscle, various cell types, including inflammatory cells and fibro-adipogenic progenitors (FAPs), contribute to tissue remodeling. To determine whether the observed MMPs activity in this study could be definitively attributed to muscle cells, an analysis of Pearson’s correlation coefficient (R) between eMyh (embryonic myosin heavy chain) expression and gelatinase (MMP-2/MMP-9) activity during muscle regeneration was performed. Immunolocalization of eMyh was performed to evaluate the progression of muscle regeneration (not shown). The signal measured as mean fluorescence intensity was not detected on day 1 post-injury. Immunolocalization of eMyh peaked at day 3 of regeneration and gradually declined in the following days in the EDL muscle. In contrast, in the Soleus muscle, the signal increased progressively, reaching its maximum level at day 14 post-injury. The Pearson’s correlation coefficient analysis revealed colocalization in both the Soleus and EDL muscles during muscle repair. In control muscles on day 1, R values were close to zero (0.00 in Soleus, 0.02 in EDL), reflecting the absence of eMyh signal at the early stage of regeneration. By day 3, R values increased (0.16 in Soleus, 0.25 in EDL), reaching their highest correlation on day 7 (0.40 in Soleus, 0.46 in EDL). These findings suggest that the observed gelatinase activity is linked to muscle cells rather than to other cell types involved in regeneration process. However, the mechanisms underlying the relationship between TGFβ1 signaling and gelatinase activity, as well as the potential differences in the signal transduction pathways responsible for the differential TGFβ1 signaling in slow- versus fast-twitch muscles, remain unknown. To determine these differences, the relationship between TGFβ1 and gelatinases in slow- and fast- muscle-derived myoblasts *in vitro* was examined.

### Inhibition of TGFβ1 signal transduction pathways impacts differently slow- and fast-twitch muscles-derived myoblast differentiation

**FIGURE 3:**
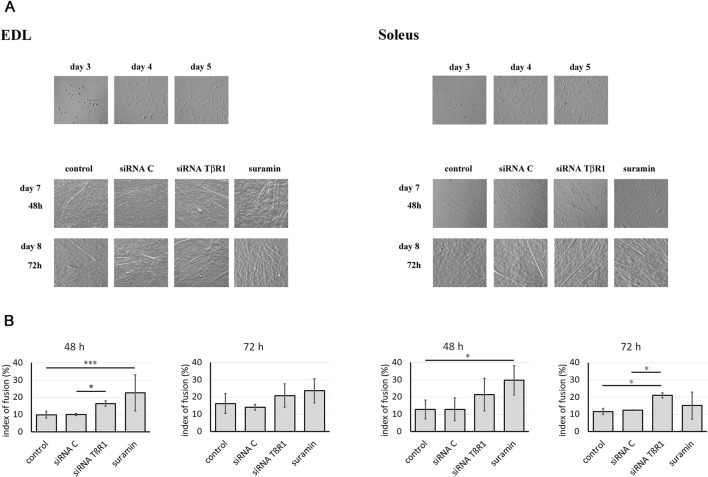
Influence of TGFβ1 signaling inhibition on myoblast differentiation. Soleus and EDL derived myoblasts were either transfected with siRNA complementary to mRNA encoding TβR1 (siRNA TβR1) or suramin treated. Untreated (control) or transfected with control siRNA (siRNA C) myoblasts were used as a control. **(A)** Morphology of Soleus and EDL derived myoblasts. The images show cell cultures on days 3, 4, and 5. The treatment with siRNA or suramin was performed on day 5, and the time-points labeled as 48 h and 72 h correspond to day 7 and 8 of culture, respectively. **(B)** Index of fusion (shown as bars) was expressed as the percentage of nuclei found in myotubes compared to the total number of nuclei. Counting was performed at 48 or 72 h after treatment on cultures stained with May Grunwald-Giemsa. Due to non-normal data distribution (Shapiro–Wilk test, *P* < 0.05), statistical analysis was performed using the Kruskal–Wallis test followed by Dunn’s multiple comparisons test. A *P* value < 0.001 was considered statistically significant. Adjusted P values are indicated as follows P < 0.05 → *; P < 0.01 → **; P < 0.001 → ***; P < 0.0001 → ****; P > 0.05 → not significant. Data are presented as mean ± SD.

To inhibit the TGFβ1 signal transduction pathways, myoblasts were transfected with siRNA complementary to mRNA encoding TβR1 or treated with suramin. Untreated myoblasts or those transfected with control siRNA were used as control. The myoblasts analyzed in this study originated from satellite cells isolated from the Soleus and EDL muscles. In control, untreated cultures, the myoblast number began to increase on day 4 following plating and continued to grow until day 8. Around day 5, the proliferation rates of Soleus- and EDL-derived myoblasts were comparable, as both populations were in the active proliferation phase. At this time-point, the cells were transfected with the appropriate siRNA or treated with suramin. The effect of TβR1 silencing was examined 48 and 72 h after treatment, what corresponded to days 7 and 8 of culture. As differentiation progressed, the first myotubes began to form. Soleus-derived myoblasts tending to differentiate earlier and generate more robust myotubes compared to those derived from EDL. On day 7, intensive myoblasts fusion was observed, when it reached 12% in Soleus and 10% in EDL-derived control, i.e., in untreated myoblasts. Downregulation of TβR1 accelerated the myoblasts fusion in Soleus-derived myoblasts (Figure 3). Seventy-two hours after transfection, the fusion index reached 22% for cells treated with siRNA complementary to the mRNA encoding TβR1, increasing significantly compared to control, untreated cells (12%). In EDL-derived myoblasts, silencing of TβR1 expression significantly affects myoblast fusion compared to control (siRNA C) 48 h after treatment, but had no significant effect on myoblast fusion 72 h after transfection. Suramin treatment of myoblasts affected only the early stages of differentiation in myoblasts derived from the EDL and Soleus muscle and did not cause significant changes 72 h after transfection (Figure 3). The progression of myoblasts differentiation was confirmed by analyzing the expression of myogenic regulatory factors (MRFs) (not shown).

### Treatment of differentiated myoblasts with TβR1 siRNA or suramin modifies the activity of MMP-9 or MMP-2 in slow-twitch muscle-derived myoblasts

**FIGURE 4:**
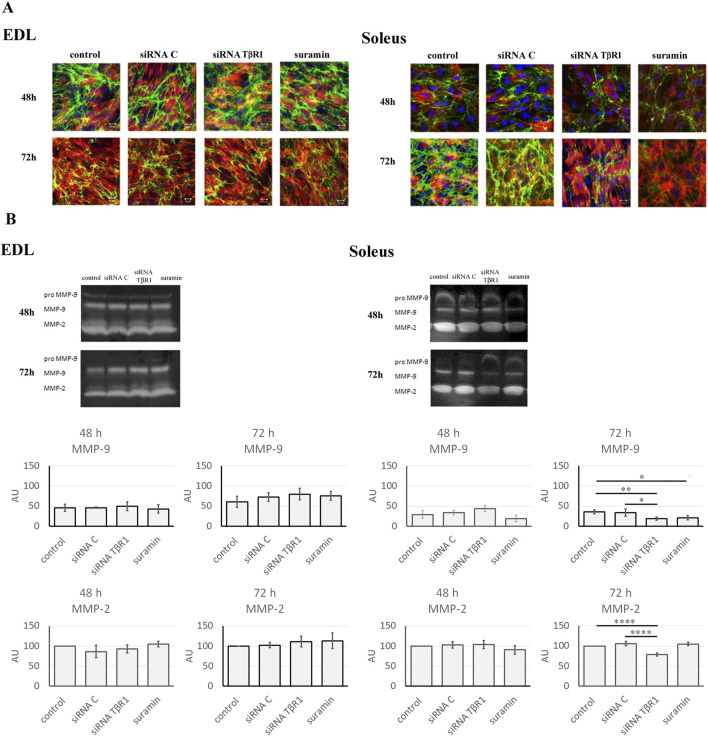
Gelatinolytic activity in Soleus and EDL derived myoblasts. Soleus and EDL derived myoblasts were either transfected with siRNA complementary to mRNA encoding TβR1 (siRNA TβR1) or suramin treated. Untreated (control) or transfected with control siRNA (siRNA C) myoblasts were used as a control. Myoblasts were analyzed at 48 or 72 h after treatment. **(A)** *in situ* zymography of *in vitro* cultured myoblasts. Gelatinolytic activity - green; actin filaments - red; nuclei–blue. Scale bar - 50 μm. **(B)** in gel zymography. Data passed Shapiro-Wilk for normal distribution (alpha = 0,05). Statistical analysis was performed using ordinary one-way ANOVA with the Holm–Šidák’s multiple comparison test. Adjusted P values from Holm-Sidak’s multiple comparisons test are indicated using the following asterisk system: P < 0.05 → *; P < 0.01 → **; P < 0.001 → ***; P < 0.0001 → ****; P > 0.05 → not significant. Data are presented as mean ± SD.

To assess the impact of inhibition of TGFβ1 signal transduction pathways on gelatinase activity, *in situ* zymography was used (Figure 4A). On day 5 of Soleus or EDL-derived myoblast culture, siRNA complementary to mRNA encoding TβR1 or treated with suramin. Based on prior analyses of culture dynamics, the time of treatment was selected as a representative time point at which Soleus- and EDL-derived myoblasts are actively proliferating. The effect of such treatment was examined after 48 or 72 h. No significant changes in gelatinolytic activity were observed in EDL-derived myoblasts at 48 or 72 h post-transfection with TβR1-targeting siRNA or suramin treatment, compared to controls (Supplementary Figure S2). However, a marked decrease in gelatinase activity was evident in Soleus-derived myoblasts treated with siRNA complementary to the mRNA encoding TβR1. Quantitative analysis of fluorescence intensity showed that the signal resulting from the gelatinase activity was noticeably reduced 72 h after transfection (Supplementary Figure S2). To further elucidate the specific MMP isoforms influenced by these treatments, in gel zymography was performed (Figure 4B). Although no significant differences in MMP-9 and MMP-2 activity were observed in EDL-derived myoblasts, a statistically significant reduction in both MMP-9 and MMP-2 activity was found in Soleus-derived myoblasts. Transfection of myoblasts with siRNA complementary to mRNA encoding TβR1 resulted in a decrease in both gelatinases at 72 h. Similarly, suramin reduced the activity of MMP-9 72 h after treatment (Figure 4B).

**FIGURE 5:**
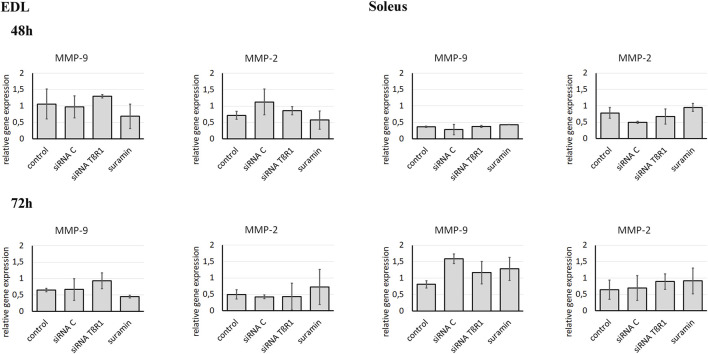
Analysis of MMP-9 and MMP-2 expression in Soleus and EDL derived myoblasts. Soleus and EDL derived myoblasts were either transfected with siRNA complementary to mRNA encoding TβR1 (siRNA TβR1) or suramin treated. Untreated (control) or transfected with control siRNA (siRNA C) myoblasts were used as a control. Analysis was performed using qRT-PCR. Relative gene expression was calculated relative to the Cq of the reference gene GAPDH. Due to non-normal data distribution (Shapiro–Wilk test, P < 0.05), statistical analysis was performed using the Kruskal–Wallis test followed by Dunn’s multiple comparisons test. A P value < 0.05 was considered statistically significant. Adjusted P values are indicated as follows: P < 0.05 → *; P < 0.01 → **; P < 0.001 → ***; P < 0.0001 → ****; P > 0.05 → not significant. Data are presented as mean ± SD.

Since downregulation of TβR1 was accompanied by a change in gelatinase activity, we focused on the impact of TβR1 downregulation on MMP-9 and MMP-2 expression (Figure 5). However, inhibition of TGFβ1 signal transduction pathways with siRNA complementary to the mRNA encoding TβR1 had no effect on the expression of MMP-9 or MMP-2 in Soleus or EDL myoblasts. Therefore, modification of the action of MMPs is associated with inhibition at the level of their activity, not with expression. To verify whether siRNA targeting TβR1 and Suramin effectively inhibited the TGFβ1 signaling pathway, Western blot analysis of Smad protein phosphorylation was performed using myoblasts isolated from both EDL and Soleus muscles (Supplementary Figure S3). In control myoblasts derived from both EDL and Soleus, phosphorylated Smad2 (P-Smad2) and Smad3 (P-Smad3) were detected, indicating that the canonical TGFβ1 pathway was active. In contrast, the levels of phosphorylated Smad2 and Smad3 were markedly reduced in EDL-derived myoblasts 48 h after siRNA TβR1 transfection or Suramin treatment. The reduction in phosphorylated Smad2 and Smad3 levels was less pronounced in Soleus-derived myoblasts. These results confirm that both siRNA-mediated knockdown of TβR1 and Suramin treatment effectively suppressed TGFβ1 signaling in muscle-derived myoblasts.

### Inhibition of TGFβ1 pathways by canonical or noncanonical inhibitors affects gelatinase activity differently in myoblasts derived from fast- and slow-twitch muscles

siRNA-driven transient downregulation of TβR1 gene expression or signal transduction inhibition by suramin can act through canonical or non-canonical pathways activated by TGFβ1-activated pathways. Therefore, we decided to specifically target these signaling pathways, hoping to more precisely block the TGFβ signaling transduction pathway. We examined the effects of canonical and noncanonical pathway inhibitors, e.g., SIS3 (Smad3 phosphorylation inhibitor), halofuginone (Smad7 activator, Smad3 phosphorylation inhibitor), U0126 and PD98059 (MEK1 inhibitors) and SB202190 (p38 MAP kinase inhibitor). Since inhibition of TGFβ1 signal transduction pathways with siRNA complementary to mRNA encoding TβR1 had no effect on the expression of MMP-9 or MMP-2 in Soleus or EDL myoblasts in subsequent experiments we focused on determining the impact of the above-mentioned inhibitors on gelatinase activity.

**FIGURE 6:**
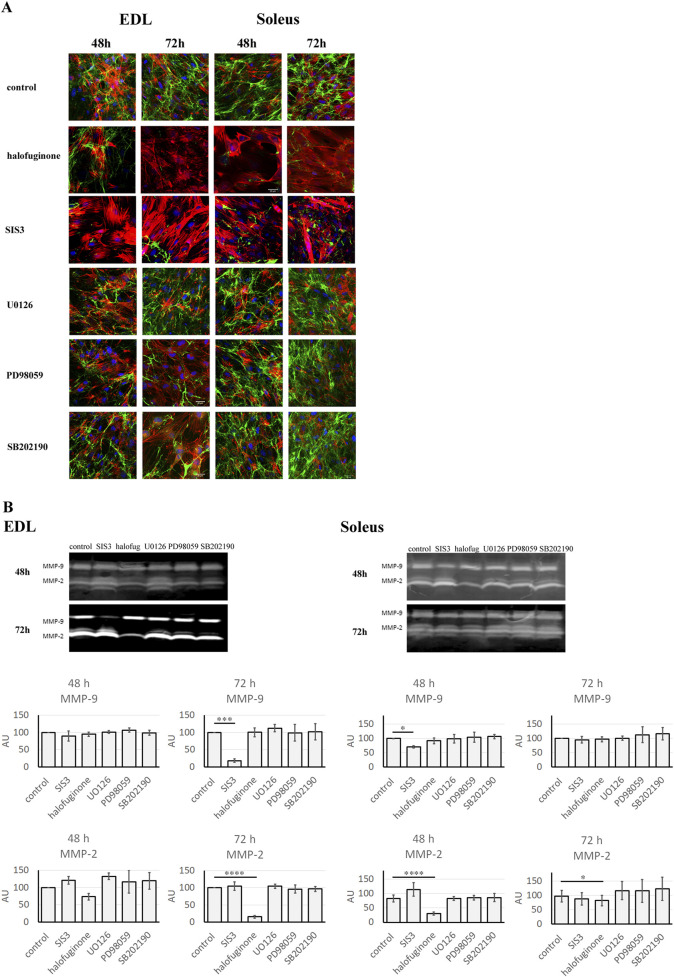
Gelatinolytic activity in Soleus and EDL derived myoblasts. Soleus and EDL derived myoblasts were treated with TGFβ signaling pathway inhibitors: SIS3, halofuginone, U0126, PD98059, or SB202190. Untreated (control) myoblasts were used as a control. Myoblasts were analyzed at 48 or 72 h after treatment. **(A)** *in situ* zymography. Gelatinolytic activity - green; actin filaments–red; nuclei–blue. Scale bar - 50 μm. **(B)** in gel zymography. For normal distribution data (P > 0.05), differences between groups were analyzed using one-way analysis of variance (ANOVA) followed by Holm–Šidák’s *post hoc* multiple comparisons test. For non-normally distributed data (P < 0.05), the nonparametric Kruskal–Wallis test was applied, followed by Dunn’s multiple comparisons test for pairwise group comparisons P < 0.05 → *; P < 0.01 → **; P < 0.001 → ***; P < 0.0001 → ****; P > 0.05 → not significant. Data are presented as mean ± SD.

Myoblasts were treated with SIS3, halofuginone, U0126, PD98059, or SB202190. Analysis of gelatinase activity was carried out 48 or 72 h after treatment. Untreated myoblasts collected at the same time points were used as a control (Figure 6A).

*In situ* zymography showed that treatment with halofuginone and SIS3 resulted in decreased gelatinase activity in both EDL and Soleus-derived myoblasts. Quantitative analysis of fluorescence intensity, performed by measuring the mean fluorescence intensity, revealed a statistically significant decrease in EDL-derived myoblasts at 72 h, as well as in Soleus-derived myoblasts (at 48 and 72 h). At the same time, U0126, PD98059, and SB202190 inhibitors had no effect on gelatinase activity in both EDL- and Soleus-derived myoblasts (Supplementary Figure S4). To quantify the changes associated with the treatment of cells with selected inhibitors more precisely, in-gel zymography was conducted. Interestingly, in gel zymography showed that the effects of inhibitors on MMP-9 and MMP-2 differed between EDL and Soleus myoblasts over time (Figure 6B). Seventy-two hours after treatment, in EDL-derived myoblasts, SIS3 reduced MMP-9 activity, while halofuginone decreased MMP-2 activity. Similarly, in Soleus myoblasts, halofuginone reduced MMP-2 activity, and SIS3 led to a reduction in MMP-9 activity. However, the response of Soleus-derived myoblasts to administered inhibitors was faster than in EDL-derived myoblasts and occurred 48 h after treatment. On the contrary, no changes in MMP-9 or MMP-2 activity were observed in EDL and Soleus-derived myoblasts treated with U0126, PD98059, SB202190 inhibitors for 48 or 72 h. This suggests that non-canonical signaling pathways may not contribute to the regulation of MMP activity in these muscle cell types.

To confirm the impact of canonical and non-canonical TGFβ signaling pathway inhibition on myoblast differentiation through the modification of MMP activity, a quantitative analysis of actin filament staining was conducted (Supplementary Figure S5). The results revealed an increased mean fluorescence intensity of actin filaments in the SIS3- and halofuginone-treated myoblasts. These results correlated with changes in MMP activity induced by both inhibitors of the TGFβ canonical signaling pathway. Statistically significant changes were observed in EDL-derived myoblasts at 72 h and in Soleus-derived myoblasts at 48 h after treatment. In contrast, treatment with U0126, PD98059, or SB202190 had no effect on the mean fluorescence intensity of actin filaments.

## Discussion

TGFβ1 (Morikawa et al., 2016; Ark et al., 2018; Ong et al., 2021; Peng et al., 2022) and MMPs (Visse and Nagase, 2003; Bassiouni et al., 2021; Biel et al., 2024) are intricately linked in various biological processes, particularly in tissue remodeling and pathological conditions, such as cancer or fibrosis development. While the role of MMPs in TGFβ1 activation is well established (Keski-Oja et al., 2004; Jenkins, 2008), the involvement of TGFβ1 signaling pathways that control MMP expression or activity remains insufficiently understood. In our study, we focused on understanding the role of TGFβ1 signaling in controlling MMP-9 and MMP-2 expression and activity during fast-twitch (EDL) and slow-twitch (Soleus) muscle regeneration. It was previously demonstrated that tissue repair after injury occurs differently in both types of muscles. The EDL muscles regenerated more effectively, whereas Soleus regeneration is delayed and often associated with fibrosis (Bassaglia and Gautron, 1995), which correlates with differences in TGFβ1 (Zimowska et al., 2009) and gelatinases (Zimowska et al., 2008) expression. Inhibiting TGFβ1 signaling through anti-TβR1 antibodies significantly improved Soleus muscle regeneration by reducing fibrosis and had no effect on EDL regeneration (Zimowska et al., 2009). Similarly, inhibition of MMP activity significantly improved Soleus muscle regeneration (Zimowska et al., 2012). Elevated levels of both MMPs (Bani et al., 2008; Li et al., 2009; Hindi et al., 2013) and TGFβ1 (Murakami et al., 1999; Salvadori et al., 2005; Noda et al., 2017) have also been reported a variety of myopathies, including fibrotic forms. Interestingly, in wooden breast myopathy, increased TGFβ1 and MMP expression coincides with collagen accumulation and ECM remodeling, indicating TGFβ1 role in promoting fibrosis through increased collagen synthesis and reduced ECM degradation (Xing et al., 2021). However, the effect of TGFβ1 on MMP expression or activity has never been investigated during muscle regeneration.

The regulation of the cellular environment is critical for effective skeletal muscle repair, influencing myoblast proliferation, fusion as well as the reconstruction of the proper innervation and vasculature of regenerating muscle. As TGFβ1 level is higher and sustained for a longer period in Soleus muscle compared to EDL muscle (Zimowska et al., 2009), its impact on the regeneration of slow-twitch muscle tissue may be more pronounced. As impaired regeneration is also accompanied by elevated levels of MMP-9 (Zimowska et al., 2008), dysregulation of this enzyme may result from excessive levels of TGFβ1. Such an increased production of MMP-2 and MMP-9 in response to TGFβ1 was found in Schwann cells (Muscella et al., 2020). It was shown that TGFβ1 promotes Schwann cell motility by upregulating MMP-2 and MMP-9 expression, facilitating ECM degradation and nerve repair (Muscella et al., 2020). Our findings suggest that a comparable TGFβ1-mediated regulation of gelatinase expression or activity may occur in regenerating skeletal muscle. *In vivo* studies have assessed the effects of TGFβ1 inhibition on MMP activity, but the complexity of the regenerating tissue hinders the identification of specific molecular mechanisms. The observed correlation between eMyh expression and gelatinase activity suggests a significant contribution of muscle cells to the regulation of MMP activity during muscle regeneration. However, this relationship requires further clarification *in vitro*. Therefore, we focused on investigating this association during myoblast differentiation under controlled *in vitro* conditions.

It was previously shown that MMP-2 and MMP-9 activity is increased by TGFβ1 treatment in a time- and dose-dependent manner in HCC1806 breast cancer cells (Kim et al., 2016). To explore this interaction in skeletal muscle, we examined the effects of modulating the TGFβ1 signaling pathway through pharmacological (suramin) and molecular siRNA-driven inhibition of the TGFβ1 receptor-activated pathway on the activity and expression of MMP-9 and MMP-2. Our results provide the first evidence that the TGFβ1 signaling pathway regulates MMPs activity. Notably, the effect varied between Soleus- and EDL-derived cells, suggesting that TGFβ1-mediated regulation of MMPs is muscle type–specific. It was previously shown that TGFβ1 regulates MMP expression and cell migration in Schwann cells via both canonical (SMAD2) and non-canonical (ERK1/2, JNK1/2, NF-κB) pathways, with MMP-2 dependent on SMAD2 and MMP-9 on the ERK1/2-JNK1/2-NF-κB pathway (Muscella et al., 2020). In our study, TβR1 siRNA and suramin effectively inhibited TGFβ1 signaling, but their broad-spectrum action may impact multiple pathways. To specify the role of the canonical TGFβ1 pathway in MMP regulation, halofuginone known as the Smad7 activator (Pines and Spector, 2015; Luo et al., 2017) and SIS3 responsible for the inhibition of Smad3 phosphorylation (Jinnin et al., 2006; Ji et al., 2018) were used. Furthermore, non-canonical pathway inhibitors were examined, e.g., affecting MAPK activity: U0126 and PD98059 (MEK1 inhibitors) and SB202190 (p38 MAP kinase inhibitor). The results have shown that MAPK inhibition had no significant effect on MMP activity in myoblasts. In contrast, blocking the canonical TGFβ1 pathway with SIS3 or halofuginone significantly reduced MMP activity in both EDL- and Soleus-derived myoblasts, although, the extent and specificity varied between muscle types. These findings highlight the critical role of the TGFβ1-Smad pathway in regulating MMPs and support the use of pathway-specific targeting strategies to enhance muscle regeneration.

We have previously shown that MMP-9 and MMP-2 play distinct roles in muscle regeneration and myoblast differentiation, with different activity profiles in fast- EDL and slow-twitch Soleus muscles (Zimowska et al., 2008). In regenerating Soleus muscle, MMP-9 levels are elevated during both myolysis and reconstruction, whereas EDL muscle shows reduced MMP-9 during myolysis and increased MMP-2 during regeneration. Similarly, *in vitro* studies revealed earlier and more pronounced MMP-9 and MMP-2 activity in Soleus-derived myoblasts compared to those from EDL. These differences align with the time-dependent effects of TGFβ1 inhibition, suggesting muscle-type-specific regulation of MMPs by TGFβ1. Additionally, TGFβ1 expression patterns during differentiation differ between these muscle types (Zimowska et al., 2009). Soleus-derived myoblasts show a strong TGFβ1 increase earlier, while EDL-derived cells exhibit delayed TGFβ1 upregulation. This may explain the observed faster response of Soleus myoblasts to TGFβ1 pathway inhibition. The earlier and more important reaction of Soleus myoblasts to inhibitors such as SIS3 or halofuginone may reflect inherent biological differences. Since fast-twitch EDL muscles are specialized for rapid contractions, slow-twitch Soleus muscles, suited for endurance and continuous activity, often display greater plasticity and regenerative potential. These intrinsic properties likely contribute to the differential sensitivity of Soleus and EDL myoblasts to TGFβ1 signaling inhibition and reflect the distinct physiological functions and metabolic profiles of these muscle types.

Our results show that SIS3 and halofuginone differentially affect MMP-9 and MMP-2 activity in Soleus- and EDL-derived myoblasts, suggesting muscle-type-specific regulatory mechanisms. Halofuginone has previously been shown to be a synthetic compound primarily known for its ability to modulate the TGFβ1 signaling pathway (Pines and Spector, 2015). It has been shown to reduce Smad3 protein levels, inhibit TGFβ-dependent Smad3 phosphorylation, and elevate Smad3 expression. Additionally, it increases the expression of the inhibitory Smad7 and reduces TGFβ receptor II protein level across various cell types, including fibroblasts, hepatic and pancreatic stellate cells, and tumor cells (Gnainsky et al., 2007; Zion et al., 2009; Spector et al., 2012; Pines and Spector, 2015; Juárez et al., 2017). Thus, its action is multifaceted. In contrast, SIS3 is a selective small-molecule inhibitor that directly targets Smad3 phosphorylation and transcriptional activity, thereby exerting a more specific and targeted inhibitory effect on Smad3-mediated signaling (Jinnin et al., 2006). Thus, halofuginone and SIS3 have different mechanisms of action, likely reflecting differences in downstream signaling pathways in Soleus and EDL-derived myoblasts. These differences may cause them to affect specific MMPs. Previous studies have shown that halofuginone inhibits MMP-2 and MMP-9 in cancer and liver tissues (Taras et al., 2006; Zcharia et al., 2012; Jin et al., 2014), while SIS3 reduces TGFβ1-induced MMP activity in HCC1806 cells (Kim et al., 2016). In our experiments, SIS3 was involved in MMP-9 activity suppression, while halofuginone reduced MMP-2 activity, suggesting distinct signaling pathways or cofactors depending on muscle type. The impact of canonical and non-canonical TGFβ signaling pathway inhibition on myoblast differentiation was confirmed through quantitative analysis of actin filament staining. The results showed an increased mean fluorescence intensity of actin filaments in the SIS3- and halofuginone-treated groups. The observed negative correlation between MMP-2 and MMP-9 activity and actin filament fluorescence intensity suggests that TGFβ inhibition affects the regulation of matrix metalloproteinase activity, and the suppression of these enzymes may facilitate cytoskeletal reorganization, potentially contributing to the promotion of myogenic differentiation. However, due to the complex regulation of MMPs, further studies are required to fully elucidate these mechanisms.

Taken together, our findings demonstrate that the TGFβ1 signaling pathway plays a role in the regulation of MMPs during myoblast differentiation. We propose that different mechanisms are active in slow- and fast-twitch-derived myoblasts to control MMP activity involving the TGFβ1 signal transduction pathway. Non-canonical signaling pathways do not appear to be regulators of MMP activity under the tested conditions, while the canonical pathway plays the main role. The different effects of inhibition of TGFβ1 on MMP-9 and MMP-2 in Soleus and EDL myoblasts highlight muscle-specific regulation. As skeletal muscle regeneration is a multiregulated process involving satellite cells, infiltrating inflammatory cells at the site of damage, and components of the extracellular matrix interacting in the damaged muscle tissue, contributing to effective muscle repair or, alternatively, to the development of fibrosis, further research is needed to elucidate the precise mechanisms underlying these interactions and to explore potential therapeutic applications for muscle-related diseases.

## Data Availability

The data presented in the study are deposited in the University of Warsaw Research Data Repository (https://danebadawcze.uw.edu.pl.pdf), accession number doi: 10.58132/MKB1SZ.

## Supplementary material

SUPPLEMENTARY FIGURE 1 Quantitative analysis of gelatinolytic activity during EDL and Soleus muscle regeneration. Gelatinolytic activity was detected in control muscles (injected with NaCl) or muscles treated with antibody against TGFβ-receptor I (TβR1 AB) analyzed at day 1, 3, 7, and 14 after the injury. Quantitative analysis was carried out by measuring the mean fluorescence intensity. Fluorescence images were acquired using a confocal laser scanning microscope (Zeiss LSM 880). The mean fluorescence intensity within each ROI was calculated by the ZEN software’s measurement tools. Data were analyzed using a two-way ANOVA with a mixed-effects model. Results are presented as mean ± SD, with individual data points representing biological replicates. Adjusted P values are indicated as follows: P < 0.05 → *; P < 0.01 → **; P < 0.001 → ***; P < 0.0001 → ****; P > 0.05 → not significant.

SUPPLEMENTARY FIGURE 2 Quantitative analysis of gelatinolytic activity in Soleus and EDL derived myoblasts. Soleus and EDL derived myoblasts were either transfected with siRNA complementary to mRNA encoding TβR1 (siRNA TβR1) or suramin treated. Untreated (control) or transfected with control siRNA (siRNA C) myoblasts were used as a control. Myoblasts were analyzed at 48 or 72 h after treatment. Quantitative analysis was carried out by measuring the mean fluorescence intensity. Fluorescence images were acquired using a confocal laser scanning microscope (Zeiss LSM 880). The mean fluorescence intensity within each ROI was calculated by the ZEN software’s measurement tools. Due to non-normal data distribution (Shapiro–Wilk test, P < 0.05), statistical analysis was performed using the Kruskal–Wallis test followed by Dunn’s multiple comparisons test. Adjusted P values are indicated as follows P < 0.05 → *; P < 0.01 → **; P < 0.001 → ***; P < 0.0001 → ****; P > 0.05 → not significant. Data are presented as mean ± SD.

SUPPLEMENTARY FIGURE 3 Western blot analysis of P-Smad in EDL and Soleus derived myoblasts. Soleus and EDL derived myoblasts were either transfected with siRNA complementary to mRNA encoding TβR1 (siRNA TβR1) or suramin treated. Untreated (control) or transfected with control siRNA (siRNA C) myoblasts were used as a control. The analysis presented was performed 48 h after siRNA transfection or Suramin treatment. Myoblasts were lysed in ice-cold buffer containing 20 mM Tris-HCl, 5 mM EGTA, 5 mM EDTA, 150 mM KCL, 1% Nonidet, 0.5% sodium deoxycholate, 0.01% leupeptin, 0.5 mM PMSF, and 10 mM β-mercaptoethanol at pH 7.5. All operations were performed on ice. Protein concentration was quantitatively determined using the Bradford Biorad Protein assay. Twenty μg of protein in Laemmli sample buffer were loaded onto a sodium dodecyl sulfate 10% acrylamide gel, transferred to PVDF membrane, and incubated with polyclonal antibodies raised against p-Smad2 (Santa Cruz) or p-Smad3 (Abcam) at final dilution 1:100 (overnight, 4°C). The blots were then incubated with peroxidase-conjugated anti-rabbit antibodies (1:2000, 1.5 h, room temperature, Abcam). The loading of gels was routinely controlled using anti-α-tubulin (Sigma). The immunoblots were visualized by chemiluminescence and exposed to film (Kodak). An image of the gel was captured using the GelDoc2000 scanner.

SUPPLEMENTARY FIGURE 4 Quantitative analysis of gelatinolytic activity in Soleus and EDL derived myoblasts. Soleus and EDL derived myoblasts were treated with TGFβ signaling pathway inhibitors: SIS3, halofuginone, U0126, PD98059, or SB202190. Untreated (control) myoblasts were used as a control. Myoblasts were analyzed at 48 or 72 h after treatment. Quantitative analysis was carried out by measuring the mean fluorescence intensity. Fluorescence images were acquired using a confocal laser scanning microscope (Zeiss LSM 880). The mean fluorescence intensity within each ROI was calculated by the ZEN software’s measurement tools. Due to non-normal data distribution (Shapiro–Wilk test, P < 0.05), statistical analysis was performed using the Kruskal–Wallis test followed by pairwise comparisons without Dunn’s correction for multiple testing. P < 0.05 → *; P < 0.01 → **; P < 0.001 → ***; P < 0.0001 → ****; P > 0.05 → not significant. Data are presented as mean ± SD.

SUPPLEMENTARY FIGURE 5 Analysis of actin fluorescence intensity. Myoblasts derived from Soleus and EDL muscles were treated with inhibitors of the TGFβ signaling pathway: SIS3, halofuginone, U0126, PD98059, or SB202190. Untreated myoblasts served as the control. Myoblasts were analyzed 48 or 72 h after treatment. Quantitative analysis of actin filament fluorescence was performed by measuring the mean fluorescence intensity. Fluorescence images were acquired using a confocal laser scanning microscope (Zeiss LSM 880). The mean fluorescence intensity within each ROI was calculated by the ZEN software’s measurement. Due to non-normal data distribution (Shapiro–Wilk test, P < 0.05), statistical analysis was performed using the Kruskal–Wallis test, followed by Dunn’s multiple comparisons test. P < 0.05 → *; P < 0.01 → **; P < 0.001 → ***; P < 0.0001 → ****; P ≥ 0.05 → not significant. Data are presented as mean ± SD.
