# Supplementary figures and images for: Differential regulation of MMP activity by TGFβ1 in fast- and slow- twitch muscle repair: insights from EDL and soleus muscle-derived myoblasts
Source: Front Cell Dev Biol. 2025 Jun 4;13:1592512. doi: 10.3389/fcell.2025.1592512 (PMC12174064; doi:10.3389/fcell.2025.1592512)

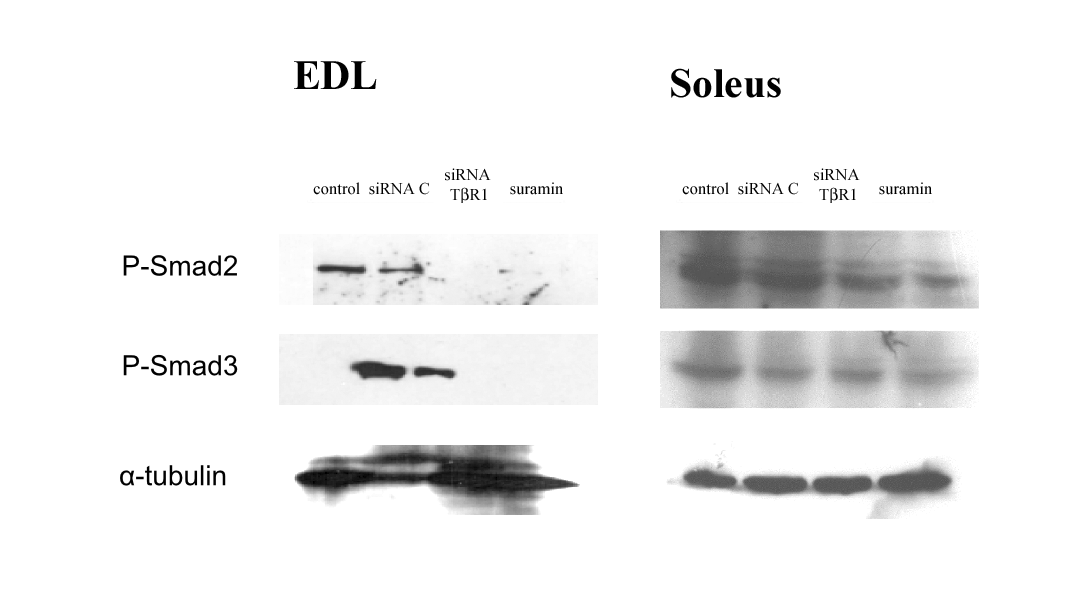

Supplement: Supplementary file 1 [file Image3.tif]

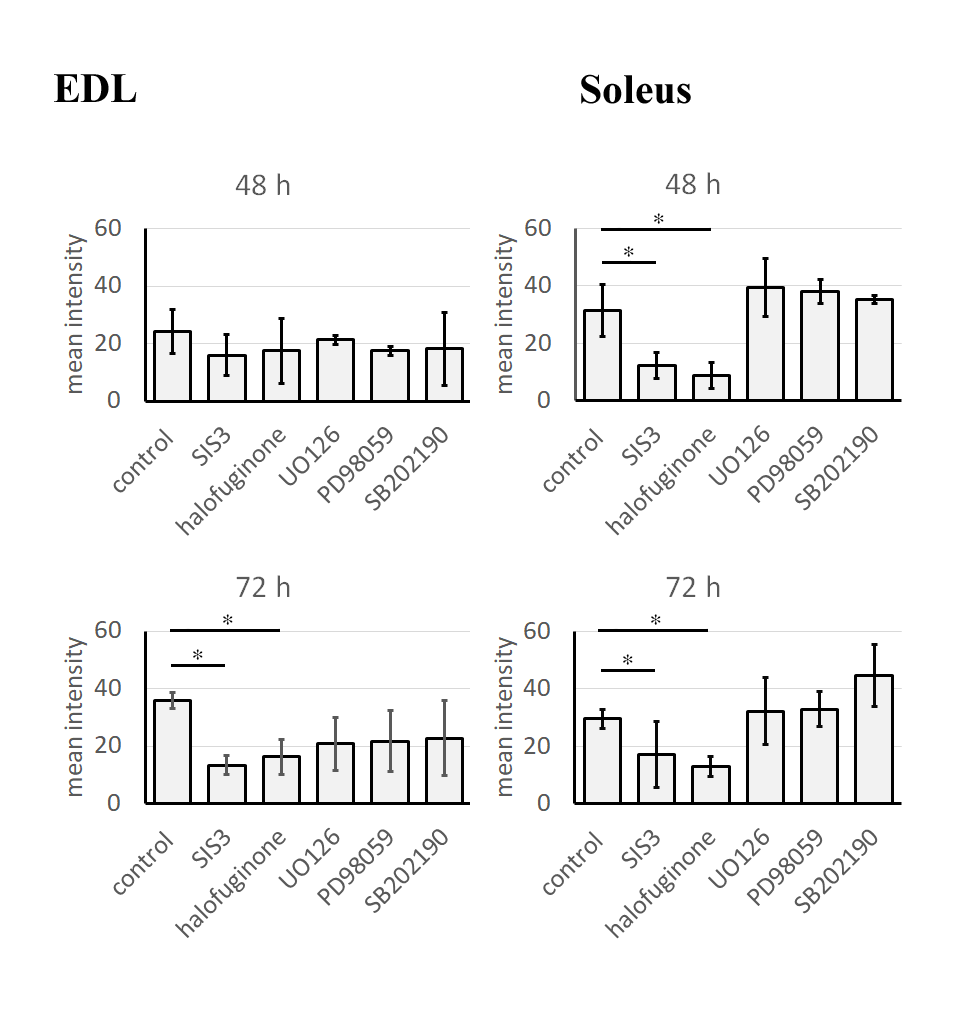

Supplement: Supplementary file 2 [file Image4.tif]

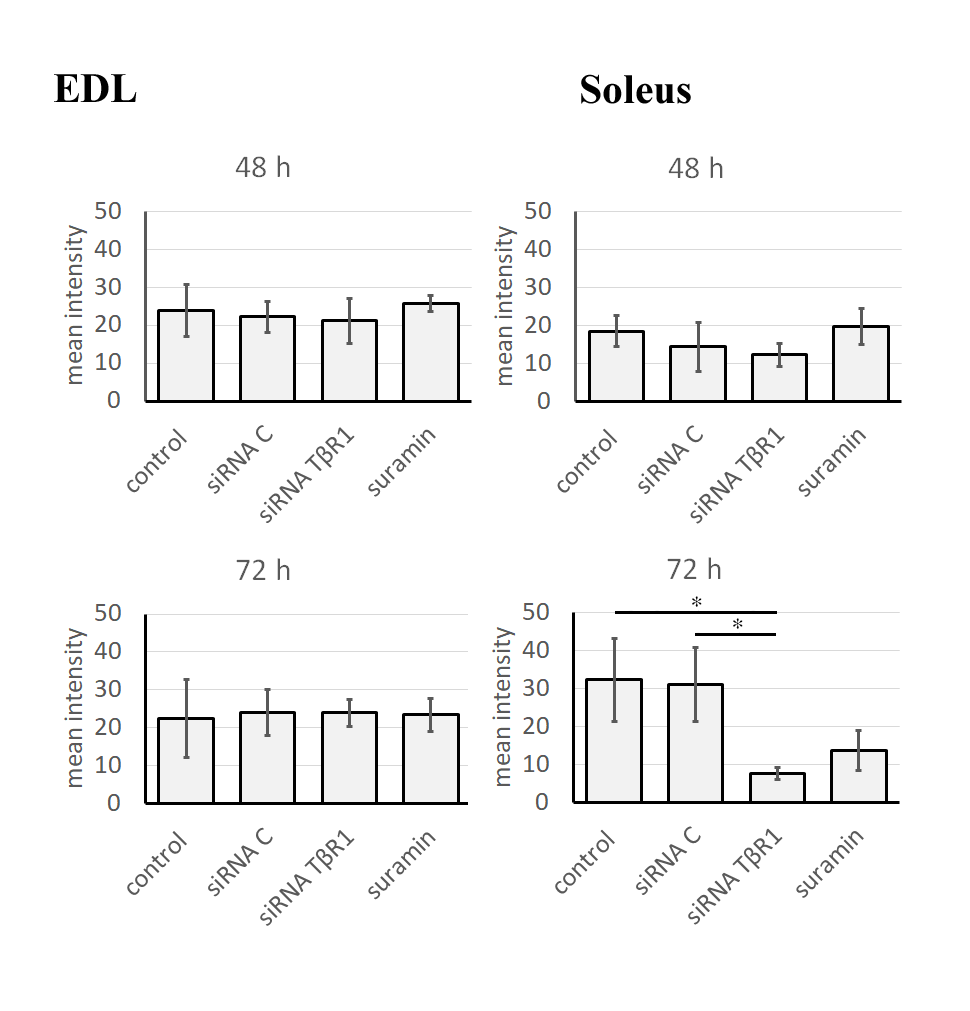

Supplement: Supplementary file 3 [file Image2.tif]

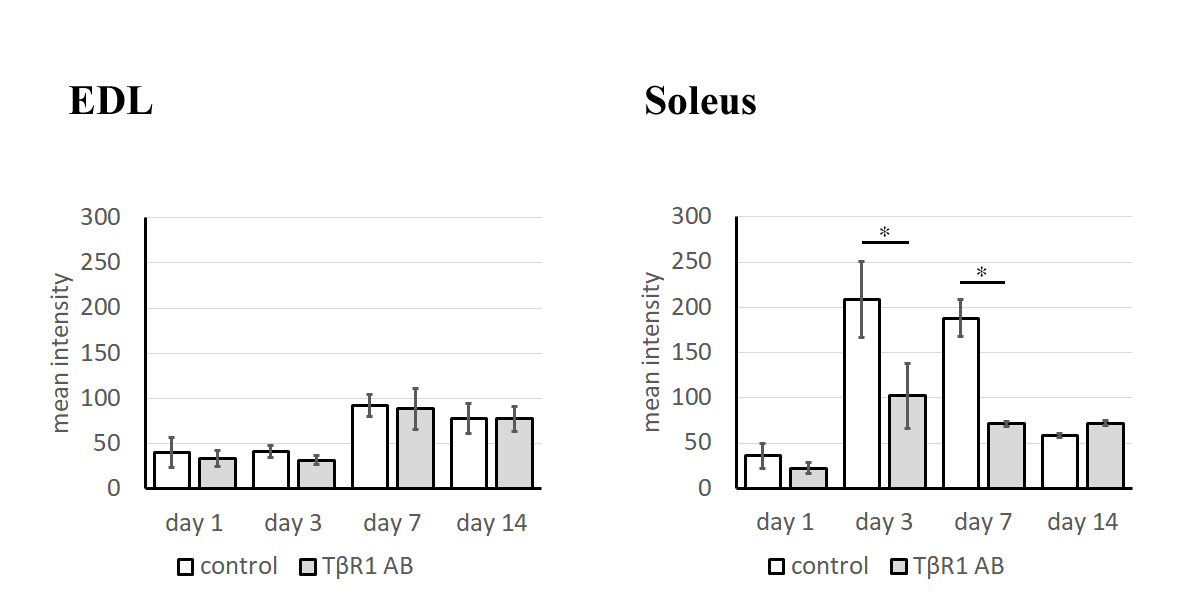

Supplement: Supplementary file 4 [file Image1.tif]

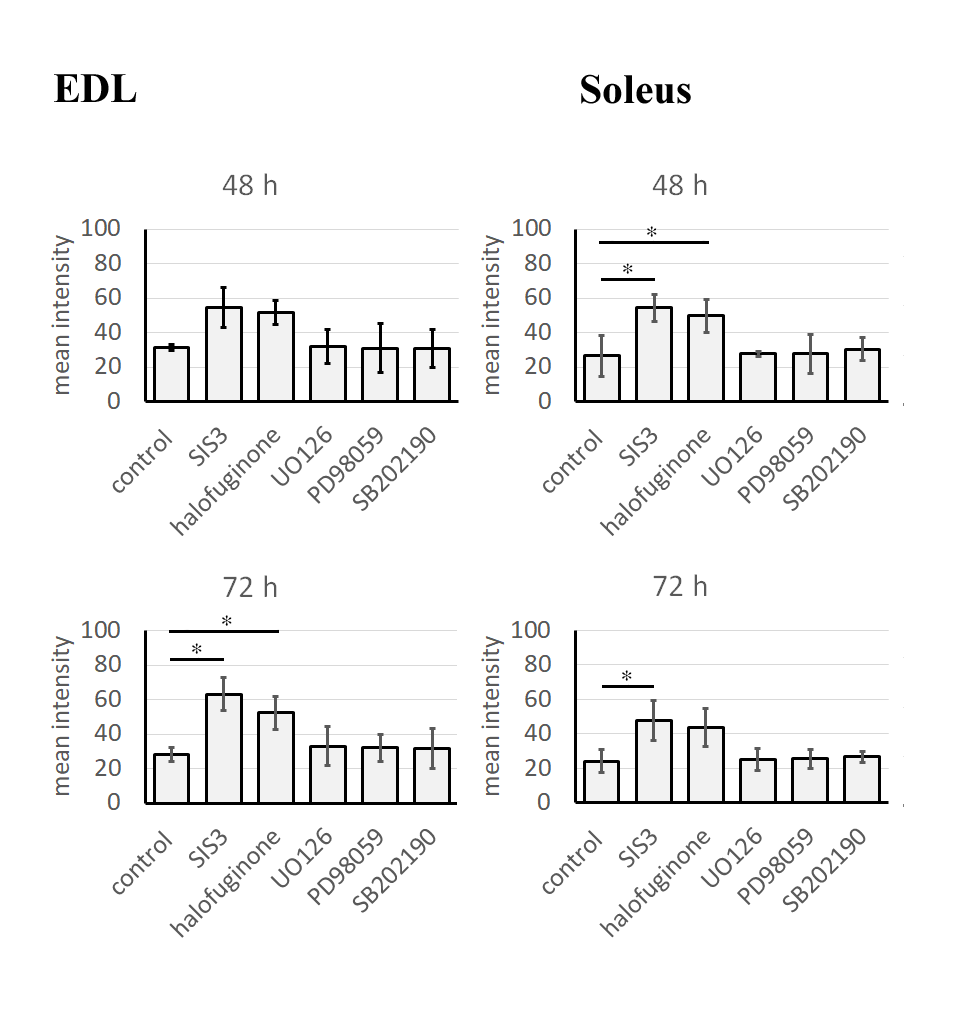

Supplement: Supplementary file 5 [file Image5.tif]
